# Crystal structure of (μ-4-hy­droxy­benzene­thiol­ato-κ^2^ *S*:*S*)bis­(μ-phenyl­methane­thiol­ato-κ^2^ *S*:*S*)bis­[(η^6^-1-isopropyl-4-methyl­benzene)­ruthenium(II)] tetra­fluorido­borate

**DOI:** 10.1107/S2056989015016953

**Published:** 2015-09-12

**Authors:** David Stíbal, Georg Süss-Fink, Bruno Therrien

**Affiliations:** a Institut de Chimie, Université de Neuchâtel, Avenue de Bellevaux 51, CH-2000 Neuchâtel, Switzerland

**Keywords:** crystal structure, ruthenium complex, piano-stool coordination geometry, thiol­ate ligands

## Abstract

The crystal structure of the dinuclear arene ruthenium title complex, [Ru_2_(C_6_H_5_OS)(C_7_H_7_S)_2_(C_10_H_14_)_2_]BF_4_, shows the two Ru^II^ atoms to be bridged by two benzyl­thio­pheno­late units and one 4-hy­droxy­thio­pheno­late unit, with the remaining three coordination sites of each Ru^II^ atom being occupied by *p*-cymene ligands, completing the typical piano-stool coordination geometry. The BF_4_^−^ counter-anion is surrounded by four cationic dinuclear complexes, showing an O—H⋯F hydrogen bond and several weak C—H⋯F inter­actions. This is the first example of an X-ray analysis of a mixed dinuclear tri­thiol­ate arene ruthenium(II) complex.

## Abstract 2

The two Ru^II^ cations, each with the characteristic piano-stool coordination geometry, are bridged by three thiol­ate ligands. The resulting dinuclear complex cation exhibits point group symmetry 1.

## Chemical context   

In the search for novel metal-based anti­cancer agents, several series of dinuclear tri­thiol­ate arene ruthenium complexes have been synthesized by our group (Gras *et al.*, 2010 ▸; Giannini *et al.*, 2012 ▸, 2013*a* ▸). The biological studies *in vitro* showed the chloride salts of these complexes to have IC_50_ values regularly in the nanomolar range, making them some of the most active ruthenium complexes found to date. The recent discovery of di­thiol­ate complexes (Ibao *et al.*, 2012 ▸) allowed us to synthesize the so-called mixed tri­thiol­ate complexes of the type [(*p*-MeC_6_H_4_^*i*^Pr)_2_Ru_2_(SCH_2_ *R*_1_)_2_(S-*p*-C_6_H_4_-*R*_2_]^+^ (*R*_1_ = C_6_H_5_, CH_2_C_6_H_5_, *p*-C_6_H_4_^*t*^Bu; *R*_2_ = H, OH, F, Br, ^*i*^Pr, ^*t*^Bu). All of the complexes were found to be highly cytotoxic against ovarian cancer cell lines A2780 and A2780cisR as chloride salts, none of them could however be crystallized and analyzed by X-ray crystallography (Giannini *et al.*, 2013*b* ▸). Herein we report the isolation and the crystal structure of the title compound, [(*p*-MeC_6_H_4_^*i*^Pr)_2_Ru_2_(SCH_2_C_6_H_5_)_2_(S-*p*-C_6_H_4_OH)]BF_4_, (I), the first reported structure of a mixed tri­thiol­ate complex.
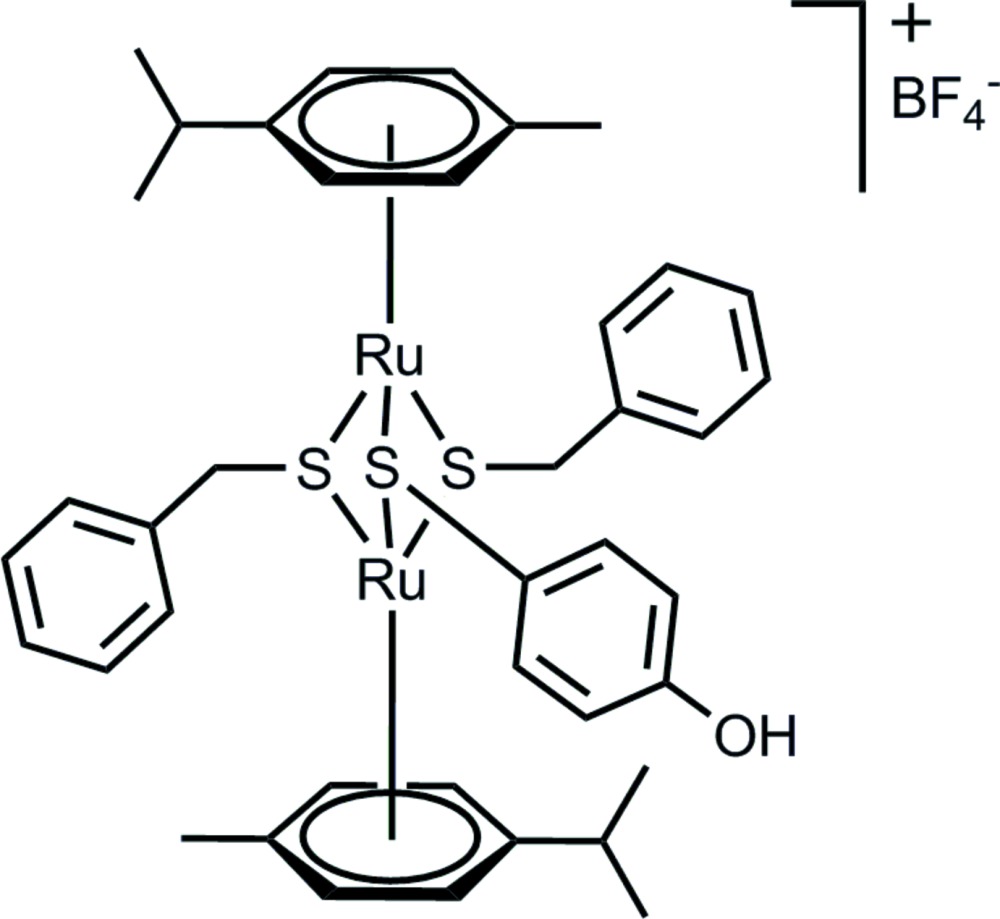


## Structural commentary   

**Figure 1:**
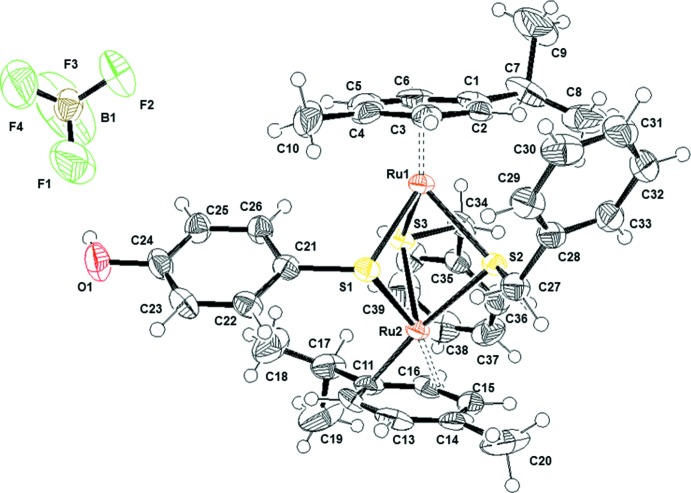
The structure of the mol­ecular components of (I). Displacement ellipsoids are drawn at the 50% probability level.

The structures of the mol­ecular components of compound (I) are presented in Fig. 1 ▸. Both Ru^II^ atoms adopt the typical piano-stool geometry with the *p*-cymene ligand being bound facially, formally occupying three coordination sites; the other three positions are occupied by two benzyl­thio­pheno­late units and one 4-hy­droxy­thio­pheno­late unit. In agreement with the electron count, there is no metal–metal bond, the Ru⋯Ru distance being 3.3632 (4) Å. The inter­atomic distances between Ru1 and S1, S2 and S3 are 2.3878 (9), 2.4023 (9) and 2.3813 (8) Å, respectively, and between Ru2 and S1, S2 and S3 2.3992 (9), 2.3991 (8) and 2.3882 (8) Å, respectively, showing that the central diruthenium tri­thiol­ate unit is not symmetric. The presence of the two bent benzyl­thiol­ate ligands forces the dinuclear arene ruthenium unit to adopt a distorted geometry – the angle between the two *p*-cymene planes (C1–C6 and C11–C16) is 6.2 (2)°. The distances between the Ru^II^ atoms and the centroids of the associated rings are 1.708 and 1.709 Å.

## Supra­molecular features   

**Figure 2:**
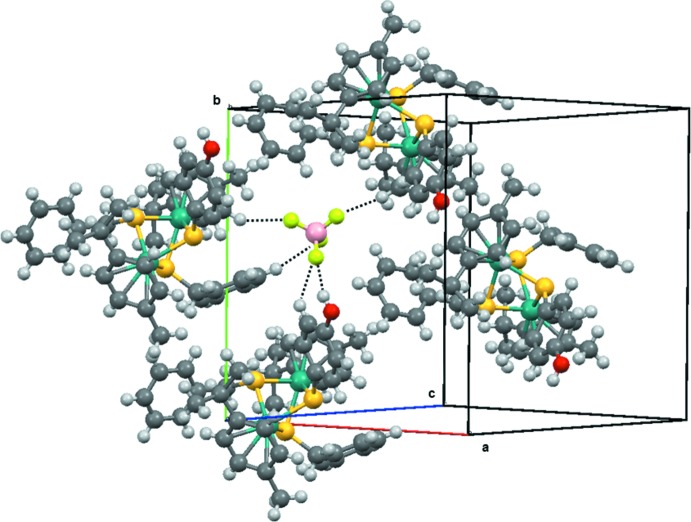
Surroundings of the BF_4_^−^ anion in the crystal packing of (I).

In the crystal packing of (I), the BF_4_^−^ anion inter­acts with the –OH group of the 4-hy­droxy­thio­pheno­late unit. In addition, weak C—H⋯F inter­actions are observed (Table 1 ▸), thus creating around the BF_4_ anion a densely packed arrangement (Fig. 2 ▸). No significant C—H⋯π or π–π stacking inter­actions are observed in the crystal structure.

## Synthesis and crystallization   

Complex (I) was obtained from the reaction of 0.127 mmol (100 mg) of the neutral di­thiol­ate precursor [(*p*-MeC_6_H_4_^*i*^Pr)_2_Ru_2_(SCH_2_C_6_H_5_)_2_Cl_2_] (Ibao *et al.*, 2012 ▸) with three equivalents of 4-hy­droxy­thio­phenol in refluxing ethanol. The product was isolated by column chromatography on silica gel, using the solvent mixture CH_2_Cl_2_/EtOH 7:1 (*v*/*v*) as eluent. The orange band was collected; the product was stirred overnight with ten equivalents of NaBF_4_ and isolated by filtration and evaporation of the solvent. X-ray quality crystals were obtained by slow diffusion of diethyl ether vapors into the solution of (I) in di­chloro­methane.

Yield: 111 mg (94%). ESI–MS (MeOH/CH_2_Cl_2_): *m*/*z* = 842.3 [*M*]^+^. ^1^H NMR (400 MHz, CDCl_3_): δ = 7.41 (*m*, 10H, SCH_2_C_6_H_5_; 2H, S-*p*-C_6_H_4_OH), 7.00 (*d*, ^3^ *J* = 8 Hz, 2H, S-*p*-C_6_H_4_OH) 5.06 [*d*, ^3^ *J* = 6.0 Hz, 2H, *p*-CH_3_C_6_H_4_CH(CH_3_)_2_], 4.94 [*d*, ^3^ *J* = 6.0 Hz, 2H, *p*-CH_3_C_6_H_4_CH(CH_3_)_2_], 4.71 [*m*, 4H, *p*-CH_3_C_6_H_4_CH(CH_3_)_2_], 3.62 (*s*, 2H, SCH_2_C_6_H_5_), 3.45 (*s*, 2H, SCH_2_C_6_H_5_), 2.04 [sept, ^3^ *J* = 6.8 Hz, 2H, *p*-CH_3_C_6_H_4_CH(CH_3_)_2_], 1.73 (*s*, 6H, *p*-CH_3_C_6_H_4_CH(CH_3_)_2_), 1.05 [*d*, ^3^ *J* = 6.8 Hz, 6H, *p*-CH_3_C_6_H_4_CH(CH_3_)_2_], 0.99 [*d*, ^3^ *J* = 6.8 Hz, 6H, *p*-CH_3_C_6_H_4_CH(CH_3_)_2_] p.p.m. ^13^C{^1^H} NMR (100 MHz,CDCl_3_): δ = 159.9, 139.9, 139.7, 133.3, 129.5, 129.2, 128.8, 128.7, 128.2, 128.1, 124.0, 117.1, 107.5, 99.7, 84.1, 83.7, 83.2, 82.0, 39.9, 39.5, 31.0, 23.1, 22.7, 18.0 p.p.m.

## Refinement   

Crystal data, data collection and structure refinement details are summarized in Table 2 ▸. All H atoms were included in calculated positions and treated as riding atoms, with C—H = 0.93 Å for C_arom_ and 0.96 Å for CH_3_, and with *U*_iso_(H) = 1.2*U*_eq_(C) or 1.5*U*_eq_(C) for methyl H atoms.

## Supplementary Material

Crystal structure: contains datablock(s) I, global. DOI: 10.1107/S2056989015016953/wm5210sup1.cif

Structure factors: contains datablock(s) I. DOI: 10.1107/S2056989015016953/wm5210Isup2.hkl

CCDC reference: 1423473

Additional supporting information:  crystallographic information; 3D view; checkCIF report

## Figures and Tables

**Table 1:** Hydrogen-bond geometry (, )

*D*H*A*	*D*H	H*A*	*D* *A*	*D*H*A*
O1H1F1	0.82	1.99	2.773(9)	161
C3H3F3^i^	0.93	2.52	3.340(11)	148
C30H30F2^i^	0.93	2.60	3.472(9)	156
C6H6F4^ii^	0.93	2.34	3.249(10)	166

Symmetry codes: (i) 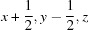; (ii) 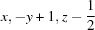.

**Table 2:** Experimental details

Crystal data	
Chemical formula	[Ru_2_(C_6_H_5_OS)(C_7_H_7_S)_2_(C_10_H_14_)_2_]BF_4_
*M*_r_	928.91
Crystal system, space group	Monoclinic, *C* *c*
Temperature (K)	173
*a*, *b*, *c* ()	15.4807(10), 14.3435(11), 17.7605(10)
()	99.435(5)
*V* (^3^)	3890.3(4)
*Z*	4
Radiation type	Mo *K*
(mm^1^)	0.99
Crystal size (mm)	0.22 0.20 0.18

Data collection	
Diffractometer	Stoe IPDS
No. of measured, independent and observed [*I* > 2(*I*)] reflections	34511, 10178, 8902
*R*_int_	0.060
(sin /)_max_ (^1^)	0.690

Refinement	
*R*[*F* ^2^ > 2(*F* ^2^)], *wR*(*F* ^2^), *S*	0.032, 0.074, 0.95
No. of reflections	10178
No. of parameters	466
No. of restraints	2
H-atom treatment	H-atom parameters constrained
_max_, _min_ (e ^3^)	0.52, 0.69
Absolute structure	Flack *x* determined using 3741 quotients [(*I* ^+^)(*I* )]/[(*I* ^+^)+(*I* )] (Parsons *et al.*, 2013 ▸).
Absolute structure parameter	0.01(2)

Computer programs: *EXPOSE*, *CELL* and *INTEGRATE* in *IPDS Software* (Stoe Cie, 2000 ▸), *SHELXS97* and *SHELXL97* (Sheldrick, 2008 ▸), *SHELXL2014* (Sheldrick, 2015 ▸) and *ORTEP-32* (Farrugia, 2012 ▸).

## supplementary crystallographic information

### Crystal data

**Table:**

[Ru_2_(C_6_H_5_OS)(C_7_H_7_S)_2_(C_10_H_14_)_2_]BF_4_	*F*(000) = 1888
*M**_r_* = 928.91	*D*_x_ = 1.586 Mg m^−^^3^
Monoclinic, *C**c*	Mo *K*α radiation, λ = 0.71073 Å
Hall symbol: C -2yc	Cell parameters from 8000 reflections
*a* = 15.4807 (10) Å	θ = 2.1–28.7°
*b* = 14.3435 (11) Å	µ = 0.99 mm^−^^1^
*c* = 17.7605 (10) Å	*T* = 173 K
β = 99.435 (5)°	Block, red
*V* = 3890.3 (4) Å^3^	0.22 × 0.20 × 0.18 mm
*Z* = 4	

### Data collection

**Table:**

Stoe IPDS diffractometer	8902 reflections with *I* > 2σ(*I*)
Radiation source: fine-focus sealed tube	*R*_int_ = 0.060
Graphite monochromator	θ_max_ = 29.4°, θ_min_ = 2.0°
Detector resolution: 0.81 pixels mm^-1^	*h* = −21→21
phi oscillation scans	*k* = −19→19
34511 measured reflections	*l* = −24→24
10178 independent reflections	

### Refinement

**Table:**

Refinement on *F*^2^	Secondary atom site location: difference Fourier map
Least-squares matrix: full	Hydrogen site location: inferred from neighbouring sites
*R*[*F*^2^ > 2σ(*F*^2^)] = 0.032	H-atom parameters constrained
*wR*(*F*^2^) = 0.074	*w* = 1/[σ^2^(*F*_o_^2^) + (0.0431*P*)^2^] where *P* = (*F*_o_^2^ + 2*F*_c_^2^)/3
*S* = 0.95	(Δ/σ)_max_ = 0.001
10178 reflections	Δρ_max_ = 0.52 e Å^−^^3^
466 parameters	Δρ_min_ = −0.69 e Å^−^^3^
2 restraints	Absolute structure: Flack *x* determined using 3741 quotients [(*I*^+^)-(*I*^-^)]/[(*I*^+^)+(*I*^-^)] (Parsons *et al.*, 2013).
Primary atom site location: structure-invariant direct methods	Absolute structure parameter: −0.01 (2)

### Special details

**Table:**

Experimental. A crystal was mounted at 173 K on a Stoe Image Plate Diffraction System (Stoe & Cie, 2000) using Mo *K*α graphite monochromated radiation. Image plate distance 100 mm, φ oscillation scans 0 - 180°, step Δφ = 1.2°, 5 minutes per frame.
Geometry. All e.s.d.'s (except the e.s.d. in the dihedral angle between two l.s. planes) are estimated using the full covariance matrix. The cell e.s.d.'s are taken into account individually in the estimation of e.s.d.'s in distances, angles and torsion angles; correlations between e.s.d.'s in cell parameters are only used when they are defined by crystal symmetry. An approximate (isotropic) treatment of cell e.s.d.'s is used for estimating e.s.d.'s involving l.s. planes.
Refinement. Refinement of *F*^2^ against ALL reflections. The weighted *R*-factor *wR* and goodness of fit *S* are based on *F*^2^, conventional *R*-factors *R* are based on *F*, with *F* set to zero for negative *F*^2^. The threshold expression of *F*^2^ > σ(*F*^2^) is used only for calculating *R*-factors(gt) *etc*. and is not relevant to the choice of reflections for refinement. *R*-factors based on *F*^2^ are statistically about twice as large as those based on *F*, and *R*- factors based on ALL data will be even larger.

### Fractional atomic coordinates and isotropic or equivalent isotropic displacement parameters (Å^2^)

**Table:**

	*x*	*y*	*z*	*U*_iso_*/*U*_eq_	
C1	0.3292 (2)	0.1913 (3)	−0.0509 (2)	0.0418 (9)	
C2	0.3924 (2)	0.1437 (2)	0.0030 (2)	0.0331 (8)	
H2	0.4249	0.0957	−0.0136	0.040*	
C3	0.4066 (2)	0.1680 (3)	0.0807 (3)	0.0374 (8)	
H3	0.4490	0.1366	0.1146	0.045*	
C4	0.3569 (3)	0.2402 (3)	0.1080 (3)	0.0465 (10)	
C5	0.2935 (3)	0.2860 (3)	0.0556 (3)	0.0528 (13)	
H5	0.2601	0.3330	0.0725	0.063*	
C6	0.2797 (3)	0.2620 (3)	−0.0221 (3)	0.0539 (13)	
H6	0.2369	0.2934	−0.0555	0.065*	
C7	0.3156 (3)	0.1726 (5)	−0.1358 (3)	0.0646 (15)	
H7	0.2531	0.1806	−0.1556	0.077*	
C8	0.3413 (5)	0.0761 (5)	−0.1566 (3)	0.085 (2)	
H8A	0.4037	0.0691	−0.1436	0.128*	
H8B	0.3238	0.0665	−0.2104	0.128*	
H8C	0.3130	0.0310	−0.1290	0.128*	
C9	0.3654 (6)	0.2436 (6)	−0.1742 (5)	0.110 (3)	
H9A	0.4271	0.2323	−0.1605	0.165*	
H9B	0.3520	0.3050	−0.1581	0.165*	
H9C	0.3489	0.2386	−0.2286	0.165*	
C10	0.3719 (3)	0.2648 (4)	0.1917 (3)	0.0667 (16)	
H10A	0.4222	0.3047	0.2030	0.100*	
H10B	0.3817	0.2088	0.2215	0.100*	
H10C	0.3213	0.2965	0.2038	0.100*	
C11	−0.0326 (3)	−0.0067 (3)	0.0892 (3)	0.0397 (10)	
C12	0.0199 (3)	−0.0321 (3)	0.1574 (3)	0.0441 (10)	
H12	0.0179	0.0032	0.2010	0.053*	
C13	0.0767 (3)	−0.1107 (3)	0.1620 (3)	0.0466 (10)	
H13	0.1105	−0.1268	0.2084	0.056*	
C14	0.0818 (3)	−0.1643 (3)	0.0962 (3)	0.0484 (11)	
C15	0.0298 (3)	−0.1365 (3)	0.0277 (3)	0.0472 (10)	
H15	0.0328	−0.1701	−0.0166	0.057*	
C16	−0.0262 (2)	−0.0602 (3)	0.0242 (3)	0.0419 (9)	
H16	−0.0601	−0.0443	−0.0222	0.050*	
C17	−0.0956 (3)	0.0766 (3)	0.0801 (3)	0.0545 (12)	
H17	−0.0907	0.1071	0.0316	0.065*	
C18	−0.0757 (4)	0.1487 (4)	0.1421 (5)	0.082 (2)	
H18A	−0.0799	0.1208	0.1905	0.123*	
H18B	−0.1170	0.1989	0.1324	0.123*	
H18C	−0.0175	0.1724	0.1430	0.123*	
C19	−0.1899 (3)	0.0414 (4)	0.0745 (6)	0.095 (3)	
H19A	−0.2025	−0.0017	0.0328	0.143*	
H19B	−0.2296	0.0932	0.0660	0.143*	
H19C	−0.1967	0.0107	0.1212	0.143*	
C20	0.1397 (3)	−0.2491 (3)	0.0989 (5)	0.091 (3)	
H20A	0.1881	−0.2428	0.1399	0.137*	
H20B	0.1613	−0.2550	0.0515	0.137*	
H20C	0.1065	−0.3037	0.1071	0.137*	
C21	0.1759 (2)	0.1523 (2)	0.2144 (2)	0.0318 (7)	
C22	0.1763 (2)	0.1270 (3)	0.2903 (2)	0.0381 (8)	
H22	0.1959	0.0680	0.3069	0.046*	
C23	0.1479 (3)	0.1880 (3)	0.3412 (2)	0.0456 (9)	
H23	0.1476	0.1698	0.3914	0.055*	
C24	0.1202 (2)	0.2753 (3)	0.3176 (2)	0.0431 (9)	
C25	0.1177 (3)	0.3026 (3)	0.2427 (2)	0.0407 (8)	
H25	0.0976	0.3617	0.2269	0.049*	
C26	0.1457 (2)	0.2409 (3)	0.1908 (2)	0.0365 (8)	
H26	0.1440	0.2589	0.1403	0.044*	
C27	0.3138 (2)	−0.1002 (3)	0.0697 (3)	0.0413 (9)	
H27A	0.3159	−0.0859	0.1234	0.050*	
H27B	0.2955	−0.1646	0.0617	0.050*	
C28	0.4040 (2)	−0.0887 (3)	0.0494 (3)	0.0382 (9)	
C29	0.4732 (3)	−0.0540 (3)	0.1000 (3)	0.0463 (10)	
H29	0.4660	−0.0401	0.1497	0.056*	
C30	0.5543 (3)	−0.0392 (3)	0.0774 (3)	0.0540 (12)	
H30	0.6005	−0.0153	0.1121	0.065*	
C31	0.5663 (3)	−0.0597 (3)	0.0048 (3)	0.0536 (11)	
H31	0.6203	−0.0487	−0.0101	0.064*	
C32	0.4971 (3)	−0.0975 (3)	−0.0476 (3)	0.0601 (13)	
H32	0.5052	−0.1129	−0.0968	0.072*	
C33	0.4171 (3)	−0.1112 (3)	−0.0250 (3)	0.0500 (11)	
H33	0.3710	−0.1358	−0.0595	0.060*	
C34	0.0879 (2)	0.1145 (3)	−0.0955 (2)	0.0406 (8)	
H34A	0.1073	0.1690	−0.1205	0.049*	
H34B	0.1209	0.0612	−0.1088	0.049*	
C35	−0.0078 (2)	0.0988 (3)	−0.1235 (2)	0.0376 (8)	
C36	−0.0411 (3)	0.0170 (3)	−0.1584 (3)	0.0511 (10)	
H36	−0.0032	−0.0302	−0.1678	0.061*	
C37	−0.1319 (4)	0.0053 (4)	−0.1796 (3)	0.0602 (13)	
H37	−0.1538	−0.0494	−0.2036	0.072*	
C38	−0.1877 (3)	0.0729 (4)	−0.1652 (3)	0.0652 (14)	
H38	−0.2478	0.0638	−0.1782	0.078*	
C39	−0.1565 (3)	0.1543 (5)	−0.1317 (3)	0.0691 (15)	
H39	−0.1950	0.2013	−0.1229	0.083*	
C40	−0.0678 (3)	0.1665 (3)	−0.1112 (3)	0.0494 (10)	
H40	−0.0472	0.2222	−0.0882	0.059*	
O1	0.0975 (2)	0.3350 (3)	0.3719 (2)	0.0593 (8)	
H1	0.0820	0.3852	0.3519	0.089*	
S1	0.22003 (5)	0.07062 (6)	0.15529 (5)	0.02976 (16)	
S2	0.23352 (5)	−0.02440 (6)	0.01262 (5)	0.02999 (17)	
S3	0.11024 (5)	0.13178 (6)	0.00909 (5)	0.02880 (17)	
Ru1	0.265363 (15)	0.136095 (17)	0.044464 (15)	0.02751 (6)	
Ru2	0.107114 (15)	−0.013494 (17)	0.074285 (15)	0.02631 (6)	
B1	0.1140 (3)	0.5937 (4)	0.2810 (3)	0.0508 (12)	
F1	0.0880 (5)	0.5128 (3)	0.3096 (4)	0.161 (3)	
F2	0.1726 (2)	0.5769 (3)	0.23242 (19)	0.0842 (11)	
F3	0.0429 (4)	0.6320 (7)	0.2444 (4)	0.196 (4)	
F4	0.1457 (4)	0.6454 (6)	0.3380 (4)	0.212 (5)	

### Atomic displacement parameters (Å^2^)

**Table:**

	*U*^11^	*U*^22^	*U*^33^	*U*^12^	*U*^13^	*U*^23^
C1	0.0331 (17)	0.046 (2)	0.049 (2)	−0.0089 (16)	0.0128 (17)	0.0188 (18)
C2	0.0272 (16)	0.0314 (17)	0.043 (2)	−0.0065 (13)	0.0120 (15)	0.0043 (15)
C3	0.0244 (15)	0.0380 (19)	0.050 (2)	−0.0071 (14)	0.0069 (15)	0.0017 (18)
C4	0.0360 (18)	0.035 (2)	0.070 (3)	−0.0164 (16)	0.0148 (19)	−0.009 (2)
C5	0.044 (2)	0.0277 (19)	0.093 (4)	−0.0083 (16)	0.030 (3)	0.002 (2)
C6	0.037 (2)	0.037 (2)	0.091 (4)	−0.0002 (16)	0.020 (2)	0.033 (2)
C7	0.044 (2)	0.104 (4)	0.048 (3)	−0.009 (3)	0.015 (2)	0.029 (3)
C8	0.111 (5)	0.102 (5)	0.041 (3)	−0.062 (4)	0.011 (3)	−0.002 (3)
C9	0.147 (7)	0.088 (4)	0.119 (6)	0.024 (4)	0.089 (5)	0.058 (4)
C10	0.048 (2)	0.073 (3)	0.082 (4)	−0.022 (2)	0.021 (2)	−0.042 (3)
C11	0.0280 (17)	0.0348 (19)	0.060 (3)	−0.0046 (14)	0.0184 (18)	0.0097 (18)
C12	0.046 (2)	0.050 (2)	0.044 (2)	−0.0096 (18)	0.0295 (19)	0.0008 (19)
C13	0.0347 (18)	0.058 (2)	0.049 (2)	−0.0050 (17)	0.0108 (17)	0.030 (2)
C14	0.0350 (19)	0.0304 (18)	0.086 (4)	−0.0031 (15)	0.029 (2)	0.017 (2)
C15	0.041 (2)	0.040 (2)	0.066 (3)	−0.0169 (17)	0.028 (2)	−0.014 (2)
C16	0.0275 (17)	0.054 (2)	0.045 (2)	−0.0154 (17)	0.0077 (16)	0.0044 (19)
C17	0.038 (2)	0.041 (2)	0.088 (4)	0.0049 (17)	0.020 (2)	0.014 (2)
C18	0.058 (3)	0.047 (3)	0.147 (6)	0.004 (2)	0.034 (4)	−0.018 (3)
C19	0.036 (2)	0.049 (3)	0.205 (8)	0.001 (2)	0.032 (4)	−0.002 (4)
C20	0.051 (3)	0.037 (2)	0.197 (8)	0.004 (2)	0.053 (4)	0.031 (4)
C21	0.0247 (14)	0.0372 (18)	0.0332 (18)	−0.0013 (13)	0.0034 (13)	0.0016 (14)
C22	0.0385 (18)	0.041 (2)	0.0360 (19)	0.0022 (15)	0.0097 (15)	0.0095 (15)
C23	0.047 (2)	0.060 (3)	0.033 (2)	0.0005 (19)	0.0154 (17)	0.0044 (18)
C24	0.0354 (19)	0.056 (2)	0.041 (2)	0.0008 (17)	0.0153 (16)	−0.0088 (18)
C25	0.0425 (19)	0.0381 (19)	0.042 (2)	0.0079 (16)	0.0078 (17)	−0.0012 (16)
C26	0.0432 (19)	0.0360 (18)	0.0303 (18)	0.0049 (15)	0.0062 (15)	0.0029 (14)
C27	0.0338 (18)	0.0351 (19)	0.058 (3)	0.0087 (15)	0.0176 (18)	0.0067 (18)
C28	0.0307 (17)	0.0327 (19)	0.054 (2)	0.0045 (14)	0.0142 (17)	0.0050 (17)
C29	0.041 (2)	0.045 (2)	0.054 (3)	0.0077 (17)	0.0079 (18)	0.0115 (19)
C30	0.0323 (19)	0.056 (3)	0.070 (3)	−0.0020 (18)	−0.002 (2)	0.014 (2)
C31	0.034 (2)	0.051 (3)	0.080 (3)	0.0052 (18)	0.022 (2)	0.006 (2)
C32	0.054 (3)	0.053 (3)	0.082 (4)	−0.005 (2)	0.038 (3)	−0.015 (3)
C33	0.040 (2)	0.047 (2)	0.067 (3)	−0.0054 (17)	0.020 (2)	−0.014 (2)
C34	0.0354 (18)	0.058 (2)	0.0292 (19)	0.0035 (16)	0.0076 (15)	0.0106 (17)
C35	0.0370 (18)	0.043 (2)	0.033 (2)	−0.0005 (15)	0.0069 (15)	0.0046 (16)
C36	0.064 (3)	0.050 (2)	0.040 (2)	0.003 (2)	0.008 (2)	−0.0050 (19)
C37	0.067 (3)	0.054 (3)	0.055 (3)	−0.022 (2)	−0.004 (2)	−0.005 (2)
C38	0.047 (2)	0.079 (4)	0.064 (3)	−0.006 (3)	−0.008 (2)	0.008 (3)
C39	0.039 (2)	0.092 (4)	0.071 (4)	0.010 (2)	−0.006 (2)	−0.015 (3)
C40	0.039 (2)	0.048 (2)	0.057 (3)	0.0047 (17)	−0.0073 (19)	−0.005 (2)
O1	0.071 (2)	0.064 (2)	0.0481 (18)	0.0128 (17)	0.0241 (16)	−0.0114 (16)
S1	0.0282 (4)	0.0300 (4)	0.0310 (4)	0.0016 (3)	0.0046 (3)	0.0047 (3)
S2	0.0257 (4)	0.0291 (4)	0.0370 (5)	−0.0027 (3)	0.0104 (3)	0.0007 (3)
S3	0.0242 (4)	0.0319 (4)	0.0307 (4)	−0.0001 (3)	0.0057 (3)	0.0069 (3)
Ru1	0.02280 (11)	0.02572 (12)	0.03464 (14)	−0.00190 (10)	0.00659 (10)	0.00523 (11)
Ru2	0.02355 (11)	0.02625 (12)	0.03065 (13)	−0.00107 (11)	0.00895 (9)	0.00414 (12)
B1	0.045 (2)	0.054 (3)	0.053 (3)	0.005 (2)	0.008 (2)	−0.008 (2)
F1	0.235 (7)	0.072 (3)	0.220 (7)	0.026 (3)	0.171 (6)	0.007 (3)
F2	0.080 (2)	0.118 (3)	0.060 (2)	0.033 (2)	0.0282 (17)	0.0103 (19)
F3	0.111 (4)	0.341 (11)	0.141 (5)	0.118 (5)	0.035 (4)	0.067 (6)
F4	0.189 (6)	0.306 (9)	0.170 (6)	−0.184 (7)	0.113 (5)	−0.171 (6)

### Geometric parameters (Å, º)

**Table:**

C1—C6	1.416 (7)	C20—H20A	0.9600
C1—C2	1.425 (6)	C20—H20B	0.9600
C1—C7	1.513 (7)	C20—H20C	0.9600
C1—Ru1	2.240 (4)	C21—C26	1.395 (5)
C2—C3	1.406 (6)	C21—C22	1.396 (5)
C2—Ru1	2.213 (3)	C21—S1	1.782 (4)
C2—H2	0.9300	C22—C23	1.382 (6)
C3—C4	1.421 (6)	C22—H22	0.9300
C3—Ru1	2.224 (4)	C23—C24	1.367 (6)
C3—H3	0.9300	C23—H23	0.9300
C4—C5	1.400 (7)	C24—O1	1.376 (5)
C4—C10	1.508 (7)	C24—C25	1.382 (6)
C4—Ru1	2.233 (4)	C25—C26	1.396 (5)
C5—C6	1.404 (8)	C25—H25	0.9300
C5—Ru1	2.196 (4)	C26—H26	0.9300
C5—H5	0.9300	C27—C28	1.508 (5)
C6—Ru1	2.189 (4)	C27—S2	1.828 (4)
C6—H6	0.9300	C27—H27A	0.9700
C7—C8	1.504 (9)	C27—H27B	0.9700
C7—C9	1.507 (8)	C28—C29	1.375 (6)
C7—H7	0.9800	C28—C33	1.406 (6)
C8—H8A	0.9600	C29—C30	1.395 (6)
C8—H8B	0.9600	C29—H29	0.9300
C8—H8C	0.9600	C30—C31	1.364 (8)
C9—H9A	0.9600	C30—H30	0.9300
C9—H9B	0.9600	C31—C32	1.408 (8)
C9—H9C	0.9600	C31—H31	0.9300
C10—H10A	0.9600	C32—C33	1.377 (6)
C10—H10B	0.9600	C32—H32	0.9300
C10—H10C	0.9600	C33—H33	0.9300
C11—C12	1.392 (7)	C34—C35	1.501 (5)
C11—C16	1.403 (6)	C34—S3	1.849 (4)
C11—C17	1.534 (6)	C34—H34A	0.9700
C11—Ru2	2.223 (4)	C34—H34B	0.9700
C12—C13	1.424 (6)	C35—C40	1.385 (6)
C12—Ru2	2.174 (4)	C35—C36	1.386 (6)
C12—H12	0.9300	C36—C37	1.405 (7)
C13—C14	1.411 (7)	C36—H36	0.9300
C13—Ru2	2.199 (4)	C37—C38	1.350 (8)
C13—H13	0.9300	C37—H37	0.9300
C14—C15	1.402 (7)	C38—C39	1.363 (8)
C14—C20	1.507 (6)	C38—H38	0.9300
C14—Ru2	2.244 (4)	C39—C40	1.372 (6)
C15—C16	1.391 (6)	C39—H39	0.9300
C15—Ru2	2.215 (4)	C40—H40	0.9300
C15—H15	0.9300	O1—H1	0.8200
C16—Ru2	2.213 (4)	S1—Ru1	2.3878 (9)
C16—H16	0.9300	S1—Ru2	2.3992 (9)
C17—C18	1.505 (8)	S2—Ru2	2.3991 (8)
C17—C19	1.532 (6)	S2—Ru1	2.4023 (9)
C17—H17	0.9800	S3—Ru1	2.3813 (8)
C18—H18A	0.9600	S3—Ru2	2.3882 (8)
C18—H18B	0.9600	B1—F4	1.286 (7)
C18—H18C	0.9600	B1—F3	1.304 (7)
C19—H19A	0.9600	B1—F1	1.355 (7)
C19—H19B	0.9600	B1—F2	1.372 (6)
C19—H19C	0.9600		
			
C6—C1—C2	117.0 (4)	C25—C26—H26	119.8
C6—C1—C7	119.4 (4)	C28—C27—S2	112.0 (3)
C2—C1—C7	123.6 (4)	C28—C27—H27A	109.2
C6—C1—Ru1	69.4 (2)	S2—C27—H27A	109.2
C2—C1—Ru1	70.3 (2)	C28—C27—H27B	109.2
C7—C1—Ru1	133.2 (3)	S2—C27—H27B	109.2
C3—C2—C1	121.4 (4)	H27A—C27—H27B	107.9
C3—C2—Ru1	71.9 (2)	C29—C28—C33	118.7 (4)
C1—C2—Ru1	72.4 (2)	C29—C28—C27	122.4 (4)
C3—C2—H2	119.3	C33—C28—C27	118.8 (4)
C1—C2—H2	119.3	C28—C29—C30	120.6 (4)
Ru1—C2—H2	128.7	C28—C29—H29	119.7
C2—C3—C4	120.6 (4)	C30—C29—H29	119.7
C2—C3—Ru1	71.1 (2)	C31—C30—C29	120.5 (4)
C4—C3—Ru1	71.7 (2)	C31—C30—H30	119.7
C2—C3—H3	119.7	C29—C30—H30	119.7
C4—C3—H3	119.7	C30—C31—C32	120.0 (4)
Ru1—C3—H3	130.0	C30—C31—H31	120.0
C5—C4—C3	118.4 (4)	C32—C31—H31	120.0
C5—C4—C10	121.5 (4)	C33—C32—C31	119.1 (5)
C3—C4—C10	120.1 (4)	C33—C32—H32	120.5
C5—C4—Ru1	70.2 (2)	C31—C32—H32	120.5
C3—C4—Ru1	71.1 (2)	C32—C33—C28	121.0 (4)
C10—C4—Ru1	129.5 (3)	C32—C33—H33	119.5
C4—C5—C6	121.0 (4)	C28—C33—H33	119.5
C4—C5—Ru1	73.0 (2)	C35—C34—S3	111.4 (3)
C6—C5—Ru1	71.1 (2)	C35—C34—H34A	109.3
C4—C5—H5	119.5	S3—C34—H34A	109.3
C6—C5—H5	119.5	C35—C34—H34B	109.3
Ru1—C5—H5	128.8	S3—C34—H34B	109.3
C5—C6—C1	121.7 (4)	H34A—C34—H34B	108.0
C5—C6—Ru1	71.6 (2)	C40—C35—C36	117.0 (4)
C1—C6—Ru1	73.3 (2)	C40—C35—C34	119.6 (4)
C5—C6—H6	119.1	C36—C35—C34	123.4 (4)
C1—C6—H6	119.1	C35—C36—C37	120.3 (4)
Ru1—C6—H6	128.2	C35—C36—H36	119.9
C8—C7—C9	109.5 (5)	C37—C36—H36	119.9
C8—C7—C1	114.1 (4)	C38—C37—C36	120.4 (4)
C9—C7—C1	109.5 (6)	C38—C37—H37	119.8
C8—C7—H7	107.8	C36—C37—H37	119.8
C9—C7—H7	107.8	C37—C38—C39	120.4 (5)
C1—C7—H7	107.8	C37—C38—H38	119.8
C7—C8—H8A	109.5	C39—C38—H38	119.8
C7—C8—H8B	109.5	C38—C39—C40	119.5 (5)
H8A—C8—H8B	109.5	C38—C39—H39	120.3
C7—C8—H8C	109.5	C40—C39—H39	120.3
H8A—C8—H8C	109.5	C39—C40—C35	122.5 (5)
H8B—C8—H8C	109.5	C39—C40—H40	118.8
C7—C9—H9A	109.5	C35—C40—H40	118.8
C7—C9—H9B	109.5	C24—O1—H1	109.5
H9A—C9—H9B	109.5	C21—S1—Ru1	114.81 (12)
C7—C9—H9C	109.5	C21—S1—Ru2	111.85 (11)
H9A—C9—H9C	109.5	Ru1—S1—Ru2	89.27 (3)
H9B—C9—H9C	109.5	C27—S2—Ru2	108.48 (13)
C4—C10—H10A	109.5	C27—S2—Ru1	110.18 (15)
C4—C10—H10B	109.5	Ru2—S2—Ru1	88.93 (3)
H10A—C10—H10B	109.5	C34—S3—Ru1	106.43 (12)
C4—C10—H10C	109.5	C34—S3—Ru2	110.79 (14)
H10A—C10—H10C	109.5	Ru1—S3—Ru2	89.68 (3)
H10B—C10—H10C	109.5	C6—Ru1—C5	37.3 (2)
C12—C11—C16	117.6 (4)	C6—Ru1—C2	66.74 (14)
C12—C11—C17	124.4 (4)	C5—Ru1—C2	78.89 (15)
C16—C11—C17	118.0 (4)	C6—Ru1—C3	78.73 (16)
C12—C11—Ru2	69.6 (2)	C5—Ru1—C3	66.47 (16)
C16—C11—Ru2	71.2 (2)	C2—Ru1—C3	36.95 (15)
C17—C11—Ru2	129.5 (3)	C6—Ru1—C4	66.98 (19)
C11—C12—C13	121.6 (4)	C5—Ru1—C4	36.83 (18)
C11—C12—Ru2	73.5 (2)	C2—Ru1—C4	67.04 (15)
C13—C12—Ru2	71.9 (2)	C3—Ru1—C4	37.19 (15)
C11—C12—H12	119.2	C6—Ru1—C1	37.27 (17)
C13—C12—H12	119.2	C5—Ru1—C1	67.45 (18)
Ru2—C12—H12	127.5	C2—Ru1—C1	37.31 (14)
C14—C13—C12	120.1 (4)	C3—Ru1—C1	67.13 (15)
C14—C13—Ru2	73.2 (2)	C4—Ru1—C1	79.82 (16)
C12—C13—Ru2	70.0 (2)	C6—Ru1—S3	93.80 (11)
C14—C13—H13	119.9	C5—Ru1—S3	103.06 (12)
C12—C13—H13	119.9	C2—Ru1—S3	145.72 (11)
Ru2—C13—H13	129.1	C3—Ru1—S3	169.43 (11)
C15—C14—C13	117.4 (4)	C4—Ru1—S3	132.82 (11)
C15—C14—C20	120.7 (5)	C1—Ru1—S3	111.14 (11)
C13—C14—C20	121.8 (5)	C6—Ru1—S1	147.03 (15)
C15—C14—Ru2	70.5 (2)	C5—Ru1—S1	113.05 (15)
C13—C14—Ru2	69.7 (2)	C2—Ru1—S1	133.72 (11)
C20—C14—Ru2	131.6 (3)	C3—Ru1—S1	104.49 (11)
C16—C15—C14	121.9 (4)	C4—Ru1—S1	95.42 (12)
C16—C15—Ru2	71.6 (2)	C1—Ru1—S1	170.98 (11)
C14—C15—Ru2	72.8 (2)	S3—Ru1—S1	77.72 (3)
C16—C15—H15	119.1	C6—Ru1—S2	134.30 (16)
C14—C15—H15	119.1	C5—Ru1—S2	171.64 (15)
Ru2—C15—H15	129.0	C2—Ru1—S2	97.38 (10)
C15—C16—C11	121.4 (4)	C3—Ru1—S2	114.82 (11)
C15—C16—Ru2	71.8 (2)	C4—Ru1—S2	148.05 (12)
C11—C16—Ru2	72.0 (2)	C1—Ru1—S2	105.05 (12)
C15—C16—H16	119.3	S3—Ru1—S2	75.75 (3)
C11—C16—H16	119.3	S1—Ru1—S2	74.95 (3)
Ru2—C16—H16	129.5	C12—Ru2—C13	38.01 (17)
C18—C17—C19	110.6 (5)	C12—Ru2—C16	66.05 (17)
C18—C17—C11	114.1 (5)	C13—Ru2—C16	78.40 (16)
C19—C17—C11	109.4 (4)	C12—Ru2—C15	78.34 (16)
C18—C17—H17	107.5	C13—Ru2—C15	66.03 (18)
C19—C17—H17	107.5	C16—Ru2—C15	36.62 (17)
C11—C17—H17	107.5	C12—Ru2—C11	36.90 (18)
C17—C18—H18A	109.5	C13—Ru2—C11	67.55 (15)
C17—C18—H18B	109.5	C16—Ru2—C11	36.87 (16)
H18A—C18—H18B	109.5	C15—Ru2—C11	66.59 (15)
C17—C18—H18C	109.5	C12—Ru2—C14	67.56 (16)
H18A—C18—H18C	109.5	C13—Ru2—C14	37.03 (19)
H18B—C18—H18C	109.5	C16—Ru2—C14	66.43 (17)
C17—C19—H19A	109.5	C15—Ru2—C14	36.65 (19)
C17—C19—H19B	109.5	C11—Ru2—C14	79.68 (14)
H19A—C19—H19B	109.5	C12—Ru2—S3	120.02 (12)
C17—C19—H19C	109.5	C13—Ru2—S3	157.02 (13)
H19A—C19—H19C	109.5	C16—Ru2—S3	99.26 (12)
H19B—C19—H19C	109.5	C15—Ru2—S3	124.53 (14)
C14—C20—H20A	109.5	C11—Ru2—S3	96.70 (10)
C14—C20—H20B	109.5	C14—Ru2—S3	160.63 (15)
H20A—C20—H20B	109.5	C12—Ru2—S2	161.04 (13)
C14—C20—H20C	109.5	C13—Ru2—S2	124.58 (12)
H20A—C20—H20C	109.5	C16—Ru2—S2	124.98 (12)
H20B—C20—H20C	109.5	C15—Ru2—S2	102.07 (11)
C26—C21—C22	118.4 (3)	C11—Ru2—S2	159.91 (13)
C26—C21—S1	124.4 (3)	C14—Ru2—S2	101.26 (10)
C22—C21—S1	117.2 (3)	S3—Ru2—S2	75.68 (3)
C23—C22—C21	121.1 (4)	C12—Ru2—S1	97.29 (12)
C23—C22—H22	119.5	C13—Ru2—S1	96.53 (12)
C21—C22—H22	119.5	C16—Ru2—S1	158.92 (12)
C24—C23—C22	119.9 (4)	C15—Ru2—S1	157.00 (14)
C24—C23—H23	120.1	C11—Ru2—S1	122.26 (13)
C22—C23—H23	120.1	C14—Ru2—S1	120.75 (14)
C23—C24—O1	117.3 (4)	S3—Ru2—S1	77.37 (3)
C23—C24—C25	120.8 (4)	S2—Ru2—S1	74.81 (3)
O1—C24—C25	121.9 (4)	F4—B1—F3	109.4 (7)
C24—C25—C26	119.6 (4)	F4—B1—F1	107.3 (6)
C24—C25—H25	120.2	F3—B1—F1	105.7 (6)
C26—C25—H25	120.2	F4—B1—F2	113.0 (5)
C21—C26—C25	120.3 (4)	F3—B1—F2	110.6 (5)
C21—C26—H26	119.8	F1—B1—F2	110.6 (5)
			
C6—C1—C2—C3	−1.9 (5)	C2—C1—Ru1—S2	−82.3 (2)
C7—C1—C2—C3	175.7 (4)	C7—C1—Ru1—S2	35.7 (5)
Ru1—C1—C2—C3	−54.9 (3)	C34—S3—Ru1—C6	63.2 (2)
C6—C1—C2—Ru1	53.1 (3)	Ru2—S3—Ru1—C6	174.80 (16)
C7—C1—C2—Ru1	−129.4 (4)	C34—S3—Ru1—C5	99.8 (2)
C1—C2—C3—C4	1.1 (5)	Ru2—S3—Ru1—C5	−148.57 (15)
Ru1—C2—C3—C4	−54.1 (3)	C34—S3—Ru1—C2	10.4 (2)
C1—C2—C3—Ru1	55.1 (3)	Ru2—S3—Ru1—C2	122.06 (17)
C2—C3—C4—C5	0.2 (5)	C34—S3—Ru1—C3	107.7 (6)
Ru1—C3—C4—C5	−53.6 (3)	Ru2—S3—Ru1—C3	−140.7 (6)
C2—C3—C4—C10	179.2 (4)	C34—S3—Ru1—C4	125.0 (2)
Ru1—C3—C4—C10	125.4 (4)	Ru2—S3—Ru1—C4	−123.41 (17)
C2—C3—C4—Ru1	53.8 (3)	C34—S3—Ru1—C1	29.23 (19)
C3—C4—C5—C6	−0.5 (6)	Ru2—S3—Ru1—C1	140.85 (12)
C10—C4—C5—C6	−179.5 (4)	C34—S3—Ru1—S1	−149.03 (15)
Ru1—C4—C5—C6	−54.6 (3)	Ru2—S3—Ru1—S1	−37.41 (3)
C3—C4—C5—Ru1	54.1 (3)	C34—S3—Ru1—S2	−71.67 (15)
C10—C4—C5—Ru1	−124.9 (4)	Ru2—S3—Ru1—S2	39.95 (3)
C4—C5—C6—C1	−0.4 (6)	C21—S1—Ru1—C6	1.2 (3)
Ru1—C5—C6—C1	−55.9 (3)	Ru2—S1—Ru1—C6	115.0 (2)
C4—C5—C6—Ru1	55.5 (3)	C21—S1—Ru1—C5	22.52 (19)
C2—C1—C6—C5	1.6 (5)	Ru2—S1—Ru1—C5	136.35 (13)
C7—C1—C6—C5	−176.1 (4)	C21—S1—Ru1—C2	119.24 (18)
Ru1—C1—C6—C5	55.1 (3)	Ru2—S1—Ru1—C2	−126.92 (14)
C2—C1—C6—Ru1	−53.5 (3)	C21—S1—Ru1—C3	92.76 (17)
C7—C1—C6—Ru1	128.8 (4)	Ru2—S1—Ru1—C3	−153.41 (11)
C6—C1—C7—C8	−156.4 (4)	C21—S1—Ru1—C4	56.07 (17)
C2—C1—C7—C8	26.1 (6)	Ru2—S1—Ru1—C4	169.91 (11)
Ru1—C1—C7—C8	−67.8 (6)	C21—S1—Ru1—S3	−76.62 (13)
C6—C1—C7—C9	80.4 (6)	Ru2—S1—Ru1—S3	37.22 (3)
C2—C1—C7—C9	−97.1 (6)	C21—S1—Ru1—S2	−154.94 (13)
Ru1—C1—C7—C9	169.0 (4)	Ru2—S1—Ru1—S2	−41.11 (3)
C16—C11—C12—C13	−1.5 (5)	C27—S2—Ru1—C6	129.7 (2)
C17—C11—C12—C13	179.6 (3)	Ru2—S2—Ru1—C6	−120.97 (15)
Ru2—C11—C12—C13	−55.8 (3)	C27—S2—Ru1—C2	65.17 (18)
C16—C11—C12—Ru2	54.4 (3)	Ru2—S2—Ru1—C2	174.49 (11)
C17—C11—C12—Ru2	−124.6 (4)	C27—S2—Ru1—C3	31.07 (18)
C11—C12—C13—C14	1.0 (6)	Ru2—S2—Ru1—C3	140.39 (12)
Ru2—C12—C13—C14	−55.6 (3)	C27—S2—Ru1—C4	7.6 (3)
C11—C12—C13—Ru2	56.5 (3)	Ru2—S2—Ru1—C4	116.9 (2)
C12—C13—C14—C15	0.4 (5)	C27—S2—Ru1—C1	102.46 (17)
Ru2—C13—C14—C15	−53.7 (3)	Ru2—S2—Ru1—C1	−148.22 (11)
C12—C13—C14—C20	−178.8 (4)	C27—S2—Ru1—S3	−149.06 (14)
Ru2—C13—C14—C20	127.1 (3)	Ru2—S2—Ru1—S3	−39.74 (3)
C12—C13—C14—Ru2	54.1 (3)	C27—S2—Ru1—S1	−68.20 (14)
C13—C14—C15—C16	−1.1 (5)	Ru2—S2—Ru1—S1	41.11 (3)
C20—C14—C15—C16	178.0 (3)	C11—C12—Ru2—C13	−132.2 (4)
Ru2—C14—C15—C16	−54.5 (3)	C11—C12—Ru2—C16	−30.0 (2)
C13—C14—C15—Ru2	53.3 (3)	C13—C12—Ru2—C16	102.2 (3)
C20—C14—C15—Ru2	−127.5 (3)	C11—C12—Ru2—C15	−66.4 (3)
C14—C15—C16—C11	0.6 (6)	C13—C12—Ru2—C15	65.8 (3)
Ru2—C15—C16—C11	−54.4 (3)	C13—C12—Ru2—C11	132.2 (4)
C14—C15—C16—Ru2	55.0 (3)	C11—C12—Ru2—C14	−103.1 (3)
C12—C11—C16—C15	0.7 (5)	C13—C12—Ru2—C14	29.0 (3)
C17—C11—C16—C15	179.7 (3)	C11—C12—Ru2—S3	57.0 (3)
Ru2—C11—C16—C15	54.3 (3)	C13—C12—Ru2—S3	−170.9 (2)
C12—C11—C16—Ru2	−53.6 (3)	C11—C12—Ru2—S2	−159.6 (3)
C17—C11—C16—Ru2	125.4 (3)	C13—C12—Ru2—S2	−27.4 (5)
C12—C11—C17—C18	20.0 (6)	C11—C12—Ru2—S1	136.5 (2)
C16—C11—C17—C18	−159.0 (4)	C13—C12—Ru2—S1	−91.3 (3)
Ru2—C11—C17—C18	−71.1 (6)	C14—C13—Ru2—C12	131.8 (4)
C12—C11—C17—C19	−104.6 (6)	C14—C13—Ru2—C16	66.1 (3)
C16—C11—C17—C19	76.4 (6)	C12—C13—Ru2—C16	−65.8 (3)
Ru2—C11—C17—C19	164.4 (5)	C14—C13—Ru2—C15	29.7 (2)
C26—C21—C22—C23	−0.4 (6)	C12—C13—Ru2—C15	−102.1 (3)
S1—C21—C22—C23	176.4 (3)	C14—C13—Ru2—C11	103.0 (3)
C21—C22—C23—C24	−1.0 (6)	C12—C13—Ru2—C11	−28.8 (3)
C22—C23—C24—O1	−176.3 (4)	C12—C13—Ru2—C14	−131.8 (4)
C22—C23—C24—C25	1.9 (6)	C14—C13—Ru2—S3	152.4 (3)
C23—C24—C25—C26	−1.4 (6)	C12—C13—Ru2—S3	20.6 (5)
O1—C24—C25—C26	176.8 (4)	C14—C13—Ru2—S2	−58.6 (3)
C22—C21—C26—C25	1.0 (5)	C12—C13—Ru2—S2	169.5 (2)
S1—C21—C26—C25	−175.6 (3)	C14—C13—Ru2—S1	−134.7 (2)
C24—C25—C26—C21	−0.1 (6)	C12—C13—Ru2—S1	93.5 (3)
S2—C27—C28—C29	115.2 (4)	C15—C16—Ru2—C12	−103.1 (3)
S2—C27—C28—C33	−62.6 (4)	C11—C16—Ru2—C12	30.0 (2)
C33—C28—C29—C30	1.6 (6)	C15—C16—Ru2—C13	−65.2 (3)
C27—C28—C29—C30	−176.2 (4)	C11—C16—Ru2—C13	67.9 (3)
C28—C29—C30—C31	−0.5 (7)	C11—C16—Ru2—C15	133.1 (4)
C29—C30—C31—C32	−1.1 (7)	C15—C16—Ru2—C11	−133.1 (4)
C30—C31—C32—C33	1.5 (7)	C15—C16—Ru2—C14	−28.3 (3)
C31—C32—C33—C28	−0.3 (7)	C11—C16—Ru2—C14	104.8 (3)
C29—C28—C33—C32	−1.1 (7)	C15—C16—Ru2—S3	138.0 (3)
C27—C28—C33—C32	176.7 (4)	C11—C16—Ru2—S3	−88.8 (2)
S3—C34—C35—C40	59.4 (5)	C15—C16—Ru2—S2	59.1 (3)
S3—C34—C35—C36	−117.3 (4)	C11—C16—Ru2—S2	−167.78 (19)
C40—C35—C36—C37	−0.1 (7)	C15—C16—Ru2—S1	−143.1 (3)
C34—C35—C36—C37	176.6 (4)	C11—C16—Ru2—S1	−10.0 (5)
C35—C36—C37—C38	−0.9 (8)	C16—C15—Ru2—C12	65.3 (3)
C36—C37—C38—C39	1.7 (9)	C14—C15—Ru2—C12	−67.9 (3)
C37—C38—C39—C40	−1.3 (9)	C16—C15—Ru2—C13	103.3 (3)
C38—C39—C40—C35	0.3 (9)	C14—C15—Ru2—C13	−30.0 (2)
C36—C35—C40—C39	0.4 (7)	C14—C15—Ru2—C16	−133.3 (4)
C34—C35—C40—C39	−176.4 (5)	C16—C15—Ru2—C11	28.5 (3)
C26—C21—S1—Ru1	17.9 (3)	C14—C15—Ru2—C11	−104.8 (3)
C22—C21—S1—Ru1	−158.7 (2)	C16—C15—Ru2—C14	133.3 (4)
C26—C21—S1—Ru2	−81.9 (3)	C16—C15—Ru2—S3	−53.2 (3)
C22—C21—S1—Ru2	101.5 (3)	C14—C15—Ru2—S3	173.49 (19)
C28—C27—S2—Ru2	−162.8 (3)	C16—C15—Ru2—S2	−134.0 (2)
C28—C27—S2—Ru1	−67.0 (3)	C14—C15—Ru2—S2	92.7 (2)
C35—C34—S3—Ru1	173.0 (3)	C16—C15—Ru2—S1	146.4 (2)
C35—C34—S3—Ru2	76.9 (3)	C14—C15—Ru2—S1	13.2 (4)
C1—C6—Ru1—C5	132.7 (4)	C16—C11—Ru2—C12	−130.4 (4)
C5—C6—Ru1—C2	−102.6 (3)	C17—C11—Ru2—C12	118.4 (6)
C1—C6—Ru1—C2	30.1 (2)	C12—C11—Ru2—C13	29.6 (3)
C5—C6—Ru1—C3	−65.8 (3)	C16—C11—Ru2—C13	−100.9 (3)
C1—C6—Ru1—C3	66.9 (3)	C17—C11—Ru2—C13	148.0 (5)
C5—C6—Ru1—C4	−28.7 (2)	C12—C11—Ru2—C16	130.4 (4)
C1—C6—Ru1—C4	104.0 (3)	C17—C11—Ru2—C16	−111.2 (6)
C5—C6—Ru1—C1	−132.7 (4)	C12—C11—Ru2—C15	102.1 (3)
C5—C6—Ru1—S3	106.6 (2)	C16—C11—Ru2—C15	−28.3 (3)
C1—C6—Ru1—S3	−120.7 (3)	C17—C11—Ru2—C15	−139.5 (5)
C5—C6—Ru1—S1	33.5 (4)	C12—C11—Ru2—C14	66.2 (3)
C1—C6—Ru1—S1	166.17 (19)	C16—C11—Ru2—C14	−64.2 (3)
C5—C6—Ru1—S2	−179.61 (19)	C17—C11—Ru2—C14	−175.4 (5)
C1—C6—Ru1—S2	−46.9 (3)	C12—C11—Ru2—S3	−133.0 (2)
C4—C5—Ru1—C6	−132.4 (4)	C16—C11—Ru2—S3	96.5 (2)
C4—C5—Ru1—C2	−66.4 (3)	C17—C11—Ru2—S3	−14.7 (5)
C6—C5—Ru1—C2	66.0 (2)	C12—C11—Ru2—S2	160.8 (3)
C4—C5—Ru1—C3	−29.8 (3)	C16—C11—Ru2—S2	30.3 (4)
C6—C5—Ru1—C3	102.7 (3)	C17—C11—Ru2—S2	−80.9 (6)
C6—C5—Ru1—C4	132.4 (4)	C12—C11—Ru2—S1	−53.8 (3)
C4—C5—Ru1—C1	−103.6 (3)	C16—C11—Ru2—S1	175.8 (2)
C6—C5—Ru1—C1	28.8 (2)	C17—C11—Ru2—S1	64.6 (5)
C4—C5—Ru1—S3	148.7 (2)	C15—C14—Ru2—C12	100.9 (3)
C6—C5—Ru1—S3	−78.9 (2)	C13—C14—Ru2—C12	−29.8 (3)
C4—C5—Ru1—S1	66.6 (3)	C20—C14—Ru2—C12	−144.9 (7)
C6—C5—Ru1—S1	−161.0 (2)	C15—C14—Ru2—C13	130.6 (3)
C3—C2—Ru1—C6	102.6 (3)	C20—C14—Ru2—C13	−115.1 (7)
C1—C2—Ru1—C6	−30.1 (3)	C15—C14—Ru2—C16	28.3 (2)
C3—C2—Ru1—C5	65.4 (3)	C13—C14—Ru2—C16	−102.4 (3)
C1—C2—Ru1—C5	−67.2 (3)	C20—C14—Ru2—C16	142.5 (7)
C1—C2—Ru1—C3	−132.7 (3)	C13—C14—Ru2—C15	−130.6 (3)
C3—C2—Ru1—C4	28.8 (2)	C20—C14—Ru2—C15	114.3 (7)
C1—C2—Ru1—C4	−103.8 (3)	C15—C14—Ru2—C11	64.4 (3)
C3—C2—Ru1—C1	132.7 (3)	C13—C14—Ru2—C11	−66.2 (3)
C3—C2—Ru1—S3	162.38 (19)	C20—C14—Ru2—C11	178.7 (7)
C1—C2—Ru1—S3	29.7 (3)	C15—C14—Ru2—S3	−16.3 (5)
C3—C2—Ru1—S1	−45.9 (3)	C13—C14—Ru2—S3	−147.0 (3)
C1—C2—Ru1—S1	−178.6 (2)	C20—C14—Ru2—S3	97.9 (7)
C3—C2—Ru1—S2	−122.1 (2)	C15—C14—Ru2—S2	−95.1 (2)
C1—C2—Ru1—S2	105.2 (2)	C13—C14—Ru2—S2	134.2 (2)
C2—C3—Ru1—C6	−66.1 (3)	C20—C14—Ru2—S2	19.1 (6)
C4—C3—Ru1—C6	66.6 (3)	C15—C14—Ru2—S1	−174.05 (19)
C2—C3—Ru1—C5	−103.2 (3)	C13—C14—Ru2—S1	55.3 (3)
C4—C3—Ru1—C5	29.5 (3)	C20—C14—Ru2—S1	−59.8 (7)
C4—C3—Ru1—C2	132.7 (4)	C34—S3—Ru2—C12	−124.07 (19)
C2—C3—Ru1—C4	−132.7 (4)	Ru1—S3—Ru2—C12	128.45 (14)
C2—C3—Ru1—C1	−28.9 (2)	C34—S3—Ru2—C13	−138.6 (3)
C4—C3—Ru1—C1	103.8 (3)	Ru1—S3—Ru2—C13	114.0 (3)
C2—C3—Ru1—S3	−111.6 (6)	C34—S3—Ru2—C16	−56.46 (17)
C4—C3—Ru1—S3	21.1 (8)	Ru1—S3—Ru2—C16	−163.94 (12)
C2—C3—Ru1—S1	147.6 (2)	C34—S3—Ru2—C15	−27.50 (18)
C4—C3—Ru1—S1	−79.7 (3)	Ru1—S3—Ru2—C15	−134.98 (13)
C2—C3—Ru1—S2	67.7 (2)	C34—S3—Ru2—C11	−93.62 (18)
C4—C3—Ru1—S2	−159.6 (2)	Ru1—S3—Ru2—C11	158.90 (13)
C5—C4—Ru1—C6	29.1 (3)	C34—S3—Ru2—C14	−15.7 (3)
C3—C4—Ru1—C6	−102.0 (3)	Ru1—S3—Ru2—C14	−123.2 (3)
C10—C4—Ru1—C6	144.1 (5)	C34—S3—Ru2—S2	67.45 (13)
C3—C4—Ru1—C5	−131.1 (4)	Ru1—S3—Ru2—S2	−40.03 (3)
C10—C4—Ru1—C5	115.0 (5)	C34—S3—Ru2—S1	144.75 (13)
C5—C4—Ru1—C2	102.5 (3)	Ru1—S3—Ru2—S1	37.27 (3)
C3—C4—Ru1—C2	−28.6 (2)	C27—S2—Ru2—C12	2.7 (4)
C10—C4—Ru1—C2	−142.5 (5)	Ru1—S2—Ru2—C12	−108.2 (4)
C5—C4—Ru1—C3	131.1 (4)	C27—S2—Ru2—C13	−17.4 (2)
C10—C4—Ru1—C3	−113.8 (5)	Ru1—S2—Ru2—C13	−128.38 (16)
C5—C4—Ru1—C1	65.8 (3)	C27—S2—Ru2—C16	−118.1 (2)
C3—C4—Ru1—C1	−65.4 (3)	Ru1—S2—Ru2—C16	130.99 (14)
C10—C4—Ru1—C1	−179.2 (5)	C27—S2—Ru2—C15	−86.5 (2)
C5—C4—Ru1—S3	−43.7 (3)	Ru1—S2—Ru2—C15	162.55 (14)
C3—C4—Ru1—S3	−174.84 (19)	C27—S2—Ru2—C11	−139.8 (3)
C10—C4—Ru1—S3	71.3 (5)	Ru1—S2—Ru2—C11	109.3 (3)
C5—C4—Ru1—S1	−122.0 (3)	C27—S2—Ru2—C14	−49.1 (2)
C3—C4—Ru1—S1	106.9 (2)	Ru1—S2—Ru2—C14	−160.00 (15)
C10—C4—Ru1—S1	−6.9 (4)	C27—S2—Ru2—S3	150.56 (16)
C5—C4—Ru1—S2	167.9 (2)	Ru1—S2—Ru2—S3	39.61 (3)
C3—C4—Ru1—S2	36.8 (4)	C27—S2—Ru2—S1	70.03 (15)
C10—C4—Ru1—S2	−77.0 (5)	Ru1—S2—Ru2—S1	−40.91 (3)
C2—C1—Ru1—C6	130.4 (4)	C21—S1—Ru2—C12	−39.83 (18)
C7—C1—Ru1—C6	−111.6 (6)	Ru1—S1—Ru2—C12	−156.38 (13)
C6—C1—Ru1—C5	−28.9 (3)	C21—S1—Ru2—C13	−78.12 (18)
C2—C1—Ru1—C5	101.6 (3)	Ru1—S1—Ru2—C13	165.33 (12)
C7—C1—Ru1—C5	−140.4 (5)	C21—S1—Ru2—C16	−3.5 (3)
C6—C1—Ru1—C2	−130.4 (4)	Ru1—S1—Ru2—C16	−120.1 (3)
C7—C1—Ru1—C2	118.0 (6)	C21—S1—Ru2—C15	−117.1 (3)
C6—C1—Ru1—C3	−101.8 (3)	Ru1—S1—Ru2—C15	126.3 (3)
C2—C1—Ru1—C3	28.7 (2)	C21—S1—Ru2—C11	−10.60 (19)
C7—C1—Ru1—C3	146.7 (5)	Ru1—S1—Ru2—C11	−127.14 (13)
C6—C1—Ru1—C4	−65.2 (3)	C21—S1—Ru2—C14	−108.01 (18)
C2—C1—Ru1—C4	65.3 (2)	Ru1—S1—Ru2—C14	135.44 (13)
C7—C1—Ru1—C4	−176.7 (5)	C21—S1—Ru2—S3	79.40 (13)
C6—C1—Ru1—S3	67.0 (3)	Ru1—S1—Ru2—S3	−37.15 (3)
C2—C1—Ru1—S3	−162.58 (19)	C21—S1—Ru2—S2	157.76 (13)
C7—C1—Ru1—S3	−44.6 (5)	Ru1—S1—Ru2—S2	41.21 (3)
C6—C1—Ru1—S2	147.2 (3)		

### Hydrogen-bond geometry (Å, º)

**Table:**

*D*—H···*A*	*D*—H	H···*A*	*D*···*A*	*D*—H···*A*
O1—H1···F1	0.82	1.99	2.773 (9)	161
C3—H3···F3^i^	0.93	2.52	3.340 (11)	148
C30—H30···F2^i^	0.93	2.60	3.472 (9)	156
C6—H6···F4^ii^	0.93	2.34	3.249 (10)	166

Symmetry codes: (i) *x*+1/2, *y*−1/2, *z*; (ii) *x*, −*y*+1, *z*−1/2.
